# Barriers and Facilitators Associated With Remote Monitoring Adherence Among Veterans With Pacemakers and Implantable Cardioverter-Defibrillators: Qualitative Cross-Sectional Study

**DOI:** 10.2196/50973

**Published:** 2023-11-21

**Authors:** Sanket S Dhruva, Merritt H Raitt, Scott Munson, Hans J Moore, Pamela Steele, Lindsey Rosman, Mary A Whooley

**Affiliations:** 1 San Francisco Veterans Affairs Medical Center San Francisco, CA United States; 2 Department of Medicine University of California, San Francisco San Francisco, CA United States; 3 Division of Cardiology Department of Specialty Care Portland Veterans Affairs Health Care System Portland, OR United States; 4 Cardiology Section Medical Service Washington DC Veterans Affairs Medical Center Washington, DC United States; 5 Division of Cardiology Department of Medicine University of North Carolina at Chapel Hill Chapel Hill, NC United States

**Keywords:** cardiac implantable electronic device, electrophysiology, pacemaker, remote monitoring, veterans, adherence

## Abstract

**Background:**

The Heart Rhythm Society strongly recommends remote monitoring (RM) of cardiovascular implantable electronic devices (CIEDs) because of the clinical outcome benefits to patients. However, many patients do not adhere to RM and, thus, do not achieve these benefits. There has been limited study of patient-level barriers and facilitators to RM adherence; understanding patient perspectives is essential to developing solutions to improve adherence.

**Objective:**

We sought to identify barriers and facilitators associated with adherence to RM among veterans with CIEDs followed by the Veterans Health Administration.

**Methods:**

We interviewed 40 veterans with CIEDs regarding their experiences with RM. Veterans were stratified into 3 groups based on their adherence to scheduled RM transmissions over the past 2 years: 6 fully adherent (≥95%), 25 partially adherent (≥65% but <95%), and 9 nonadherent (<65%). As the focus was to understand challenges with RM adherence, partially adherent and nonadherent veterans were preferentially weighted for selection. Veterans were mailed a letter stating they would be called to understand their experiences and perspectives of RM and possible barriers, and then contacted beginning 1 week after the letter was mailed. Interviews were structured (some questions allowing for open-ended responses to dive deeper into themes) and focused on 4 predetermined domains: knowledge of RM, satisfaction with RM, reasons for nonadherence, and preferences for health care engagement.

**Results:**

Of the 44 veterans contacted, 40 (91%) agreed to participate. The mean veteran age was 75.3 (SD 7.6) years, and 98% (39/40) were men. Veterans had been implanted with their current CIED for an average of 4.4 (SD 2.8) years. A total of 58% (23/40) of veterans recalled a discussion of home monitoring, and 45% (18/40) reported a good understanding of RM; however, when asked to describe RM, their understanding was sometimes incomplete or not correct. Among the 31 fully or partially adherent veterans, nearly all were satisfied with RM. Approximately one-third recalled ever being told the results of a remote transmission. Among partially or nonadherent veterans, only one-fourth reported being contacted by a Department of Veterans Affairs health care professional regarding not having sent a remote transmission; among those who had troubleshooted to ensure they could send remote transmissions, they often relied on the CIED manufacturer for help (this experience was nearly always positive). Most nonadherent veterans felt more comfortable engaging in RM if they received more information or education. Most veterans were interested in being notified of a successful remote transmission and learning the results of their remote transmissions.

**Conclusions:**

Veterans with CIEDs often had limited knowledge about RM and did not recall being contacted about nonadherence. When they were contacted and troubleshooted, the experience was positive. These findings provide opportunities to optimize strategies for educating and engaging patients in RM.

## Introduction

Cardiovascular implantable electronic devices (CIEDs: pacemakers and implantable cardioverter-defibrillators [ICDs]) are life-saving devices that provide heart rhythm therapy for patients at risk or with malignant brady- or tachyarrhythmias. CIEDs also generate important diagnostic information, which can be transmitted to clinicians through remote monitoring (RM). RM is strongly recommended by the Heart Rhythm Society (class 1, level of evidence A) and is the standard of care for all patients with CIEDs [1,2]. This is because RM has been demonstrated in randomized clinical trials and large observational studies to improve several important patient-centered outcomes, including reducing mortality [3-5], hospitalizations [4,6,7], and inappropriate ICD shocks [8]. Studies have also demonstrated that RM is associated with high levels of patient acceptance and satisfaction [9-11]. However, this research has only studied patients who are engaged in RM [9-11].

Unfortunately, adherence to RM is suboptimal. Within the Department of Veterans Affairs (VA), the largest US health system performing RM, caring for more than 60,000 veterans with CIEDs who are monitored centrally by the Veterans Affairs National Cardiac Device Surveillance Program (VANCDSP), fewer than one-third of veterans had complete adherence to scheduled transmissions over a 2-year period [12]. For patients to achieve the benefits of RM, they must be adherent to sending RM transmissions. Additionally, patients with wireless devices should ideally be consistently and continuously connected to their transmitter [2]. Ideally, patients would be informed before their CIED implantation about the purpose and benefits of RM, counseled about the importance of adherence, and provided directions about how to activate and send transmissions [2]. Patients must also be educated about steps to troubleshoot challenges with RM [2]. This ideal intervention of education, counseling, and directions may not uniformly occur because of the stress of device placement, or it may not be provided at a level individualized to patient comprehension [2].

To improve RM adherence among patients with CIEDs, we must first understand the reasons for nonadherence. Although previous research has quantitatively examined RM adherence, data about patient perspectives are limited to a single focus group study of 9 patients from 1 county in the Midwestern US [13]. To better understand patient perspectives, we conducted structured telephone interviews about potential barriers and facilitators to RM adherence with adherent and nonadherent veterans who were followed by the VANCDSP.

## Methods

### Veteran Population

Using the VANCDSP database of all veterans with CIEDs who had agreed to participate in RM as of October 23, 2020, we created this study’s sample. Veteran contact information was identified through the Veterans Affairs Corporate Data Warehouse. According to best practices for qualitative methods, our goal was to select a representative sample of veterans to understand their perspectives about barriers and facilitators associated with RM, and we continued this study until we reached saturation of information to ensure adequate data [14].

Veterans were stratified into 3 groups based on their adherence to RM transmissions over the past 2 years: fully adherent (≥95%), partially adherent (≥65% but <95%), and nonadherent (<65%). Each time that a veteran sent an RM transmission, the veteran was considered adherent for the past number of days equivalent to their transmission interval, plus an additional 10 days in order to provide a buffer for any brief delays in transmission [12]. Thus, adherence was determined based on the past 631 days (since nearly all veterans have a 90-day transmission window, and an additional 10-day buffer leads to 100 fewer days compared with the 730 days in a 2-year period).

Among the entire population of veterans who had agreed to participate in RM, we randomly selected veterans from each of the 3 groups (fully, partially, and nonadherent) in a 2:6:3 ratio, respectively, through purposive sampling [15]. First, we included a limited number of veterans who were fully adherent; as our focus was to understand challenges with RM adherence, we did not anticipate learning as much about barriers, but we wanted to have some data from these veterans. Our primary focus was veterans who were partially adherent to RM (as this group of veterans comprises most of the nonadherent veterans), and, thus, we sampled 3 times as many of these veterans in this study. Finally, we know that there are also many patients who are supposed to be engaged in RM but are nonadherent; accordingly, we included this group of veterans, but half as many as those who were partially adherent. Saturation was reached more quickly in the group of fully adherent veterans. We included veterans with both wireless-capable CIEDs and those who must manually send remote transmissions; this latter group receives a postcard reminder from the VANCDSP before their scheduled transmission date.

### Structured Interview Guide Development and Testing

The structured interview guide was developed to learn about veteran experiences with care for CIEDs. In developing the interview guide, we sought input from clinicians with expertise in RM and researchers with expertise in qualitative methods. The structured interview guide used a fixed order and number of questions with predetermined categorical answer choices. A few questions allowed for open-ended responses to dive deeper into themes. The interview guide was pretested on 3 randomly selected veterans to determine acceptability, improve clarity, and fine-tune length.

### Interview Domains

Veteran interviews covered 4 predetermined domains: knowledge, satisfaction, reasons for nonadherence, and preferences for health care engagement. The first domain asked all veterans about their understanding of RM and its benefits. The second domain asked veterans who were fully or partially adherent about their perspectives, satisfaction, and adherence to RM. The third domain was limited to veterans who were partially or nonadherent to RM; these questions asked about reasons for nonadherence, contact with the health care system or CIED manufacturer, and reminders about missed transmissions. The final CIED-related domain asked questions about preferences for engagement around home monitoring, including stopping in-person clinic visits, confirmation of transmission success, and learning the results of remote transmissions. We also asked veterans about demographics, social determinants of health, and location of care. Veterans could decline to answer any questions.

### Interview Protocol

All veterans were mailed a letter in November 2020 stating that they would be reached by phone to understand their experiences and perspectives of RM technology as well as possible barriers. They were also provided with contact information if they wanted to schedule an interview.

Approximately 1 week after the letters were mailed, veteran contact began. Veterans were reached at one of their 2 primary numbers within the electronic health record, one of which is usually a mobile phone number. Attempts were made to reach veterans a minimum of 3 times, leaving a message after the first attempt with a call-back number.

The structured interviews were conducted by 1 author (SM), who has experience conducting telephone interviews with veterans. Once saturation across responses was reached, no further interviews were conducted. During the interview, veterans were also provided with education about RM, and those who were not actively transmitting were provided reference information to support starting or restarting remote transmissions.

### Data Analysis

Study data were analyzed deductively using content analysis within the 4 study domains. The data are presented using descriptive statistics. Where available, quotations are used to illustrate themes.

### Ethical Considerations

This project did not constitute research. In accordance with the VA’s Office of Research & Development Program Guide: 1200.21, “VHA (Veterans Health Administration) Operations Activities That May Constitute Research,” data were collected as part of a quality improvement study to assess and improve the quality of RM care for veterans with CIEDs and did not require institutional review board approval. Veterans consented to participation; no compensation was provided.

## Results

Overall, we contacted 44 veterans (6 fully adherent, 28 partially adherent, and 10 nonadherent). A total of 2 veterans declined to be interviewed, and another 2 could not be reached despite multiple attempts. We concluded participant recruitment after reaching saturation.

Among the 40 veterans interviewed (6 fully adherent, 25 partially adherent, and 9 nonadherent—of whom 7 had never sent an RM transmission), the mean age for veterans was 75.3 (SD 7.6) years, and 98% (39/40) were men (Table 1). A total of 30 veterans (75%) self-reported White race, 4 (10%) Black or African American, 3 (8%) American Indian or Alaskan Native, 3 (8%) other, and 3 (8%) declined to answer. A total of 27 veterans (68%) were married, and 1 (2%) reported difficulty with housing.

**Table 1:** Characteristics of interviewed veterans receiving cardiovascular implantable electronic device care within US Department of Veterans Affairs, October 2020.

Characteristics			Fully adherent (n=6)		Partially adherent (n=25)		Nonadherent (n=9)		Total (n=40)
Age (years), mean (SD)			78.2 (6.7)		76.3 (8.1)		70.5 (4.3)		75.3 (7.6)
Male sex, n (%)			6 (100)		24 (96)		9 (100)		39 (98)
**Race, n (%)**									
	White	5 (83)		19 (76)		6 (67)		30 (75)	
	Black or African American	0 (0)		2 (8)		2 (22)		4 (10)	
	Asian	0 (0)		0 (0)		0 (0)		0 (0)	
	American Indian or Alaskan Native	0 (0)		2 (8)		1 (11)		3 (8)	
	Native Hawaiian or other Pacific Islander	0 (0)		0 (0)		0 (0)		0 (0)	
	Other	1 (17)		2 (8)		0 (0)		3 (8)	
	Declined to answer	0 (0)		2 (8)		1 (11)		3 (8)	
**Highest education attained, n (%)**									
	Less than high school	2 (33)		6 (24)		1 (11)		9 (22)	
	High school degree or completion of general educational development test	0 (0)		3 (12)		1 (11)		4 (10)	
	Some college or associate degree	2 (33)		12 (48)		4 (44)		18 (45)	
	Bachelor’s degree	1 (17)		3 (12)		2 (22)		6 (15)	
	Higher than a bachelor’s degree	1 (17)		1 (4)		0 (0)		2 (5)	
	Declined to answer	0 (0)		0 (0)		1 (11)		1 (2)	
**Marital status, n (%)**									
	Married	4 (67)		18 (72)		5 (56)		27 (68)	
	Living with a partner	1 (17)		1 (4)		1 (11)		3 (8)	
	Widowed, separated, or single	1 (17)		6 (24)		2 (22)		9 (22)	
	Declined to answer	0 (0)		0 (0)		1 (11)		1 (2)	
**Housing difficulty, n (%)**									
	No difficulty with housing	6 (100)		24 (96)		9 (100)		39 (98)	
	Difficulty with housing	0 (0)		1 (4)		0 (0)		1 (2)	
**CIED^a^ information, n (%)**									
	Pacemaker	2 (33)		15 (60)		5 (56)		22 (55)	
	Implantable cardioverter-defibrillator	4 (67)		10 (40)		4 (44)		18 (45)	
	Duration of time (years) CIED has been in place	4.3 (3.1)		4.8 (3.0)		3.4 (2.1)		4.4 (2.8)	
	Number of CIED generators in veteran’s history	2.3 (0.8)		1.4 (0.7)		1.3 (0.5)		1.6 (0.7)	
**Wireless versus manual transmission, n (%)**									
	Wireless-capable generator	5 (83)		15 (60)		7 (78)		30 (75)	
	Manual transmission only	1 (17)		10 (40)		2 (22)		10 (25)	
**Cardiology care location, n (%)**									
	All cardiology care within VA^b^	6 (100)		23 (92)		8 (89)		37 (92)	
	Some cardiology care provided outside VA	0 (0)		2 (8)		1 (11)		3 (8)	
**Overall health care location, n (%)**									
	At least half of the health care is provided within VA	6 (100)		24 (96)		9 (100)		39 (98)	
	Less than half of health care is provided within VA	0 (0)		1 (4)		0 (0)		1 (2)	

^a^CIED: cardiovascular implantable electronic device.

^b^VA: Department of Veterans Affairs.

Of the 40 veterans, a total of 22 (55%) had pacemakers, and 18 (45%) had ICDs. A total of 30 (75%) CIEDs were wireless-capable, while 10 (25%) were manual transmission only. The veterans had been implanted with their current CIED for an average of 4.4 (SD 2.8) years and had a mean of 1.6 (SD 0.7) generators in their history. Of the 40 respondents, 37 (92%) received all their cardiology care within VA, and all but one received at least half of their health care within VA.

### Domain 1: Understanding of Remote Monitoring and Clinical Benefits

Of the 40 veterans, 23 (58%) reported that home monitoring had been discussed with them, and 5 (12%) were not sure. Among these 23 veterans, only 5 (22%) recalled learning about RM at the initial implant, with 14 (61%) learning about it at follow-up for in-person CIED checks (Table 2). The person who discussed RM with the veteran was a physician for 4 (17%), a nurse for 7 (30%), a CIED manufacturer representative for 5 (22%), and unknown for 7 respondents (30%). An additional 2 veterans reported learning about RM by reading a pamphlet. Only 8 (35%) veterans were accompanied by a friend, family member, or caregiver when RM was initially discussed with them; the majority of partially adherent or nonadherent veterans reported being unaccompanied. Of the 23 veterans with whom RM had been discussed, 20 (87%) reported being satisfied or very satisfied with this discussion.

**Table 2:** Veteran-reported characteristics of remote monitoring education among interviewed veterans receiving cardiovascular implantable electronic device care within US Department of Veterans Affairs who recalled being informed about remote monitoring.

Characteristics		Fully adherent (n=5), n (%)	Partially adherent (n=11), n (%)	Nonadherent (n=7), n (%)	Total^a^ (n=23), n (%)
**Time of** **veteran’s** **education about remote monitoring**					
	At initial implant	2 (40)	2 (18)	1 (14)	5 (22)
	At follow-up for in-person CIED^b^ checks	2 (40)	8 (73)	4 (57)	14 (61)
	At other times	0 (0)	1 (9)	2 (29)	3 (13)
	Unknown	1 (20)	0 (0)	0 (0)	1 (4)
**Remote monitoring educator**					
	Physician	2 (40)	0 (0)	2 (29)	4 (17)
	Nurse	0 (0)	4 (36)	3 (43)	7 (30)
	CIED manufacturer representative	2 (40)	2 (18)	1 (14)	5 (22)
	Unknown	1 (20)	5 (45)	1 (14)	7 (30)
**Social support at remote monitoring education**					
	Accompanied by friend, family member, or caregiver	3 (60)	4 (36)	1 (14)	8 (35)
	Unaccompanied	1 (20)	6 (55)	4 (57)	11 (48)
	Not sure or unknown	1 (20)	1 (9)	2 (29)	4 (17)
**Satisfaction with remote monitoring education**					
	Satisfied or very satisfied	4 (80)	10 (91)	6 (86)	20 (87)
	Not very satisfied	1 (20)	1 (9)	1 (14)	3 (13)

^a^An additional 17 veterans did not recall being informed about remote monitoring.

^b^CIED: cardiovascular implantable electronic device.

Only 18 (45%) of the 40 veterans thought that they had a good understanding of RM. However, when these 18 veterans were asked an open-ended question to describe their understanding, some potential misconceptions were identified. A veteran said, “machine (remote transmitter) will buzz, call (name of clinician) at the VA.” Other veterans did have an understanding:

"Every 90 days, transmitter reads ICD, transmits to Boston Scientific. They decode and send results to (the) doctor."

When these 40 veterans were asked to describe the clinical benefits of RM, 19 (48%) reported detection of abnormal rhythms and 5 (12%) reported detection of device malfunction. However, 8 (20%) veterans were unable to report any benefits. Of the 40 veterans, 33 (82%) recognized that it was safer to be participating in RM than not participating, while 4 (10%) felt that it was the same, and 3 (8%) declined to answer.

### Domain 2: Perspectives and Satisfaction With Remote Monitoring

Among the 31 fully or partially adherent veterans, a total of 27 (87%) were satisfied with RM (Table 3). Of these 31 veterans, a total of 28 (90%) stated that they would recommend RM to other veterans with a CIED. When these veterans were asked an open-ended question as to why they would recommend RM, they provided a variety of reasons, most commonly that it provided “peace of mind” and a sense of security, as well as the need for fewer in-person visits.

**Table 3:** Veteran satisfaction with remote monitoring among interviewed veterans receiving cardiovascular implantable electronic device care within US Department of Veterans Affairs who were fully or partially adherent to remote monitoring.

		Fully adherent (n=6), n (%)	Partially adherent (n=25), n (%)	Total^a^ (n=31), n (%)
**Veteran’s** **satisfaction with remote monitoring**				
	Satisfied	6 (100)	21 (84)	27 (87)
	Not sure	0 (0)	4 (16)	4 (13)
**Veteran** **remote monitoring referral disposition**				
	Would recommend remote monitoring	6 (100)	22 (88)	28 (90)
	Not sure	0 (0)	3 (12)	3 (10)
**Veteran** **notification of any transmission results**				
	Notified	2 (33)	9 (36)	11 (35)
	Not notified	3 (50)	16 (64)	19 (61)
	Not sure	1 (17)	0 (0)	1 (3)

^a^An additional 9 veterans were nonadherent.

Of these 31 veterans, 11 (35%) reported having ever been told the results of a remote transmission. Only 18 (58%) veterans had an idea of the next steps if their RM transmitter was not working; approximately half said that they would contact their VA clinic and the other half would contact the manufacturer of their CIED.

### Domain 3: Adherence to Remote Monitoring

Of the 25 veterans who were partially adherent to RM, when asked about possible barriers to adherence, a total of 3 (12%) reported forgetting about monitoring, and 3 (12%) reported losing a monitor (Table 4). A total of 2 veterans reported preferring in-person visits, and none reported other concerns. No veterans reported concerns about privacy or not knowing how to engage in RM.

**Table 4:** Communication and barriers to remote monitoring among interviewed veterans receiving cardiovascular implantable electronic device care within US Department of Veterans Affairs who were partially adherent or nonadherent to remote monitoring.

			Partially adherent (n=25), n (%)		Nonadherent (n=9), n (%)		Total^a^ (n=34), n (%)
**Barriers to remote monitoring**							
	Do not know how	0 (0)		4 (44)		4 (12)	
	Do not recall being informed about remote monitoring	0 (0)		4 (44)		5 (15)	
	Privacy	0 (0)		1 (11)		1 (3)	
	Prefer in-clinic visits	2 (8)		2 (22)		4 (12)	
	Difficulties with use (either patient-specific constraints, disabilities, or technology difficulties)	0 (0)		1 (11)		1 (3)	
	Forgetting about remote monitoring	3 (12)		2 (22)		5 (15)	
	Losing monitor	3 (12)		0 (0)		3 (9)	
**Veteran contacted about missed remote monitoring transmission**							
	Contacted	6 (24)		4 (44)		10 (29)	
	Not contacted	18 (72)		5 (56)		23 (68)	
	Not sure	1 (4)		0 (0)		1 (3)	
**Veteran-manufacturer communication about remote monitoring**							
	Called manufacturer	14 (56)		3 (33)		17 (50)	
	Positive experience when called manufacturer	12 (86)		2 (67)		14 (82)	
	Negative experience when called manufacturer	2 (14)		1 (33)		3 (18)	
	Have not called manufacturer	10 (40)		6 (67)		16 (47)	
	Not sure	1 (4)		0 (0)		1 (3)	

^a^An additional 6 veterans were fully adherent.

Among these 25 veterans, only 6 (24%) reported being contacted by a VA health care professional regarding not having sent a transmission, and the same number recalled being offered help in transmitting. A total of 14 (56%) veterans had called the manufacturer of their CIED about RM, and all but 2 reported a positive experience; one of them asked for a new remote transmitter but reported that the request was declined, and the other replied that they were unable to reach anyone.

Among 9 veterans who were nonadherent, the barriers identified were not knowing how to transmit (n=4), not recalling being informed about RM (n=4), preferring in-person visits to RM (n=2), forgetting (n=2), privacy concerns (n=1), and difficulty with using a home monitor (n=1).

Of these 9 veterans, only 4 reported that they had been contacted by a VA health care professional about not sending a transmission. However, a total of 5 veterans felt that they would feel more comfortable engaging if they received more information or education; all 5 preferred to learn from VA clinicians, and an additional 2 were amenable to informational postcards. Of these 9 veterans, a total of 3 had called the manufacturer of their CIED; of which 2 reported positive experiences, while 1 reported that no solution was possible because of a lack of cell coverage. Of these 9 veterans, a total of 3 were not sure about their interest in starting RM, and 1 was not interested in starting RM, stating, “If it’s my time, it’s my time.”

### Domain 4: Additional Possibilities With Remote Monitoring

When all 40 veterans were offered the hypothetical possibility of stopping routine in-person CIED evaluations in favor of an RM-only approach, a total of 10 (25%) were interested in the possibility, while 25 (62%) were not, another 4 (10%) were not sure, and 1 (2%) declined to answer (Table 5). A majority of the veterans who were nonadherent were interested in this option.

**Table 5:** Interest in remote monitoring engagement among interviewed veterans receiving cardiovascular implantable electronic device care within US Department of Veterans Affairs.

		Fully adherent (n=6), n (%)	Partially adherent (n=25), n (%)	Nonadherent (n=9), n (%)	Total (n=40), n (%)
**Interest in complete substitution of remote monitoring for in-person visits**					
	Interested	1 (17)	4 (16)	5 (56)	10 (25)
	Not interested	4 (67)	19 (76)	2 (22)	25 (62)
	Not sure	1 (17)	2 (8)	1 (11)	4 (10)
	Declined to answer	0 (0)	0 (0)	1 (11)	1 (2)
**Interest in receiving a transmission reminder**		5 (83)	22 (88)	6 (67)	33 (82)
**Preferred format for remote monitoring transmission reminder**					
	SMS text message	2 (40)	4 (18)	4 (67)	10 (30)
	Email	1 (20)	6 (27)	0 (0)	7 (21)
	Mobile app	0 (0)	0 (0)	0 (0)	0 (0)
	Phone call	1 (20)	7 (32)	2 (33)	10 (30)
	Other: letter	1 (20)	0 (0)	0 (0)	1 (3)
	Multiple combinations	0 (0)	5 (23)	0 (0)	5 (15)
**Interest in successful transmission notification**		3 (50)	16 (64)	6 (67)	25 (62)
**Interest in learning remote monitoring results**		6 (100)	21 (84)	6 (67)	33 (82)
**Level of detail interested about remote monitoring results**					
	Normal or abnormal	6 (100)	15 (71)	5 (83)	26 (79)
	All device details	0 (0)	6 (29)	1 (17)	7 (21)
**Preferred format for learning remote monitoring results**					
	SMS text message	2 (33)	4 (19)	2 (33)	8 (24)
	Email	1 (17)	8 (38)	0 (0)	9 (27)
	Mobile app	0 (0)	3 (14)	1 (17)	4 (12)
	Letter or phone call	2 (33)	4 (19)	2 (33)	8 (24)
	Multiple options	1 (17)	2 (10)	1 (17)	4 (12)

Of the 40 veterans, a total of 25 (62%) were interested in a smartphone or tablet app notifying them of a successful transmission, and 24 (60%) had a smartphone or tablet. And 33 (82%) veterans, including majorities in all 3 categories, were willing to receive a reminder if they had missed their transmission by at least 3 days. A total of 34 respondents (all of those who said “yes” and an additional veteran who was “not sure”) reported their preferred mechanism: SMS text messaging (n=12), email (n=11), phone call (n=13), and n=1 each through a letter or a mobile app.

A total of 33 (82%) veterans were interested in learning the results of their remote transmissions; of these, a total of 26 (79%) wanted to know just if the transmission was normal or abnormal, while 7 (21%) were interested in all device details. When these 33 veterans were asked the mechanism through which they would like to learn these results, 8 (24%) stated SMS text message, 9 (27%) email, 4 (12%) mobile app, 8 (24%) other and reported that they wanted either a letter or phone call, and 5 (15%) mentioned multiple options.

Among the 13 veterans who needed to send manual transmissions, a total of 10 (77%) were interested in electronic reminders through either SMS text message, email, or both. Similarly, among the 27 veterans with wireless CIEDs, a total of 22 (81%) were interested in a reminder the day before their automatic scheduled transmission, and some of these veterans were also interested in a phone call or letter reminder.

## Discussion

### Principal Results

In this qualitative study of veterans with CIEDs, we found that most veterans reported being satisfied with RM, but they often had limited understanding about the need for and clinical benefits of RM. Some veterans did not recall receiving counseling about RM. Among veterans who were not fully adherent to RM, few recalled being contacted by clinicians about nonadherence. Nonadherent veterans welcomed the opportunity to learn more about RM and engage in monitoring. These findings are important because they demonstrate gaps in veterans’ knowledge about RM and opportunities to support veterans in increasing RM engagement so that they can achieve the many clinical outcome benefits of RM that lead to its strong professional society recommendation [1,2].

### Comparison With Previous Work

Previous single-center survey and interview research has shown that patients have limited understanding about their CIED [16,17]. This study extends these findings to a larger population and asked more specific questions about barriers and facilitators to RM. A focus group study found that patients not engaged in RM did not understand RM or have confidence in their ability to send a transmission [13], and we add more detail to these findings in a larger number of patients. This is also the first study of veterans’ perspectives and understanding of RM.

A previous survey of patients in CIED clinics found variation in the amount of detail patients request about their remote transmissions, ranging from most patients being interested in learning about battery life to a smaller proportion being interested in sensing and impedance data [16]. To date, only 1 pilot study of 10 patients has provided granular CIED data directly to patients; overall, patients appreciated access to the data but had questions about its interpretation [18]. This study demonstrates that veterans are usually interested in learning at least if a remote transmission has been received, whether it is normal or abnormal, and, less frequently, additional details.

### Patient Education at Device Implant

To ensure minimal knowledge gaps about RM among patients, the standard of care should be patient education before device implant, as recommended by the Heart Rhythm Society [1,2]. In this study, fewer than 15% of veterans recalled learning about RM at CIED placement. However, veterans were interviewed an average of almost 5 years after the placement of their current CIED. Thus, it is possible that they may not have retained that information given the stress and often overwhelming nature of hospitalization and CIED placement, as well as accompanying factors such as sedation. Therefore, the importance of RM should be reinforced in the outpatient setting and through a variety of other strategies that are individualized to patients, such as pamphlets or digital information made available through patient portals, to ensure that patients understand the multiple benefits and need to engage in RM. Including family members, when able, could also help support patients in monitoring. The inaccurate or incomplete understanding of the benefits of RM that we found for some patients is also likely to be addressed if they receive accurate information before, during, and after the implant; it is possible that some of these knowledge gaps may also lead to suboptimal adherence.

### Addressing Nonadherence to Remote Monitoring

Our findings also demonstrate that patients should be informed about nonadherence. The Heart Rhythm Society gives a Class 1 recommendation for device clinics to have an established process with dedicated clinic staff to facilitate reconnection [2]. However, clinicians usually have competing priorities, such as addressing alerts [19] and the overall deluge of transmissions [20]; thus, adequate staffing is imperative [2]. Although troubleshooting RM nonadherence through patient contact can take significant amounts of time in busy clinical schedules [21,22], RM reduces the need for in-person visits [23,24] and is associated with significant cost savings [7]. Therefore, in addition to established patient benefits, these clinician efforts are worthwhile; however, given busy clinical schedules, these ideally would be supported by additional strategies to inform patients about lost connectivity or missed transmissions. Most veterans in this study reported positive experiences receiving help from CIED manufacturers over the phone, and clinicians may just need to refer patients to manufacturers for assistance. Veterans were also amenable to a variety of, primarily digital, methods to alert them about a missed transmission.

### Context for Improving Remote Monitoring Adherence

Addressing these gaps in patient education and addressing nonadherence is particularly important because the number of patients implanted with CIEDs has been growing globally [25]; more than 350 per 100,000 Medicare beneficiaries received a CIED-related procedure in 2019 [26]. Continuous RM is also recommended if patients have a CIED component under safety advisory; the number of safety advisories has been increasing in recent years and RM is necessary to allow quicker detection and response [2]. Furthermore, during the COVID-19 pandemic, RM was strongly recommended by professional societies with the goal of minimizing unnecessary in-person visits [27,28]. Similarly, the VHA has placed increasing emphasis on digital care [29]. Achieving these goals for the increasing number of veterans with CIEDs will require ensuring patients are empowered with knowledge about RM through appropriate counseling and reinforcement of information, as appropriate.

### Further Opportunities for Improving Remote Monitoring Care

We also found that veterans were interested in learning about their remote transmissions—both if a transmission was successfully received and varying levels of details about the transmissions. In 2019, the Heart Rhythm Society issued a Call to Action about transparent sharing of digital health data, including data from CIEDs [30], and this was reinforced by a Class IIa recommendation in the 2023 Heart Rhythm Society Expert Consensus that the results of all remote transmissions be shared with patients, based on preferences for content, mode of communication, and clinic workflows [2]. Sharing this information could also help reduce additional patient-initiated transmissions [31]. This trend follows the larger goal of providing patients with increased access to their own health care data, which has been supported by legislation and regulation and is hoped to empower patients to better manage their health care [32]. Notification if a patient with a wireless device is connected and transmitting is now available for some Bluetooth-capable CIEDs, but not all veterans have a smartphone or tablet [33]. This means that while communicating CIED data to patients is important, research must delineate the relevant parameters and provide assurance that patients can comprehend the findings; if so, this holds the potential to maximize the clinical benefits of RM [2].

### Limitations

Our findings should also be considered within the context of their limitations. First, this study is limited to 40 randomly selected veterans followed by the VA and may not generalize to other populations. However, as there are more than 60,000 veterans with CIEDs followed by VA, these findings generalize to a large population, and we had a 91% response rate among veterans who we attempted to reach. Additionally, patients outside VA may face financial burdens for participating in RM. Second, this study population was enriched for veterans with intermittent or low adherence and may not reflect the views and perspectives of more adherent veterans, although veterans who are not fully adherent are those for whom understanding barriers and facilitators is most important. Third, social desirability bias may have led participants to provide answers that are inconsistent with their true viewpoints. Future studies can directly address patient viewpoints, such as through a validated questionnaire about satisfaction with RM [34].

### Conclusion

Among a population of veterans with CIEDs enriched for those intermittently or nonadherent, we found that veterans often had limited understanding of RM. Most veterans did not recall being contacted about nonadherence, but when they were contacted and troubleshooted, they found the experience to be positive. These findings demonstrate important opportunities to engage patients in RM, thereby improving both their quality of care and clinical outcomes.

## Abbreviations

CIED: cardiovascular implantable electronic device
ICD: implantable cardioverter-defibrillator
RM: remote monitoring
VA: Department of Veterans Affairs
VANCDSP: Veterans Affairs National Cardiac Device Surveillance Program
VHA: Veterans Health Administration

## Data Availability

Data sharing is not applicable to this article, as no research data sets were generated or analyzed. The results describe qualitative patient interviews that were conducted for quality improvement purposes.
